# Deep breath out: molecular survey of selected pathogens in blow and skin biopsies from North Atlantic cetaceans

**DOI:** 10.1186/s12917-025-05152-6

**Published:** 2025-12-18

**Authors:** Helena Costa, Per Ramstedt, Myrthe Bergsma, Eve Jourdain, Zoë Morange, Pierre Blévin, Charla J. Basran, Marianne H. Rasmussen, Terence P. Dawson, Harriet Y. Dawson, Seán A. O’Callaghan, Prabhugouda Siriyappagouder, Jorge M. O. Fernandes, Audun H. Rikardsen, Courtney A. Waugh

**Affiliations:** 1 Faculty of Biosciences and Aquaculture, Nord University, Universitetsalléen 11, Bodø, 8026 Norway; 2 Department of Arctic and Marine Biology, UiT The Arctic University of Norway, Hansine Hansens veg 18, Tromsø, 9019 Norway; 3 Norwegian Orca Survey, Breivikveien 10, Andenes, NO-8480 Norway; 4 Akvaplan-niva AS, Fram Centre, Hjalmar Johansens gate 14, Tromsø, 9007 Norway; 5 Húsavík Research Centre, University of Iceland, Hafnarstétt 3, 640, Húsavík, Iceland; 6 Department of Geography, King’s College London, Strand, London, WC2R 2LS UK; 7 The Royal (Dick) School of Veterinary Studies, The University of Edinburgh, Easter Bush Campus, Midlothian, EH25 9RG UK; 8 Marine and Freshwater Research Centre, Atlantic Technological University Galway City, Old Dublin Road, Galway, H91 T8NW Ireland; 9 Department of Renewable Marine Resources, Institute of Marine Sciences (ICM-CSIC), Barcelona, 08003 Spain; 10 Norwegian Institute of Nature Research, Fram Centre, Hjalmar Johansens gate 14, Tromsø, 9007 Norway

**Keywords:** Infectious disease, Drone, Morbillivirus, Herpesvirus, Influenza, Brucella, Norway, Iceland, Cape verde

## Abstract

**Background:**

Cetacean morbillivirus, herpesvirus, avian influenza virus (AIV) and *Brucella* spp. have been linked to numerous cetacean strandings in the Northeast (NE) Atlantic. Yet, their prevalence in free-living cetaceans remains insufficiently investigated, particularly in northern regions.

**Methods:**

Between 2016 and 2025, humpback whales (*Megaptera novaeangliae*), sperm whales (*Physeter macrocephalus*) and, opportunistically, fin whales (*Balaenoptera physalus*) and a long-finned pilot whale (*Globicephala melas*), were sampled in two foraging grounds in northern Norway (Skjervøy and Andenes), in Iceland and in Cape Verde. Blow samples (*n* = 76), skin biopsies (*n* = 45), and organ samples from one stranded pilot whale were collected and screened for cetacean morbillivirus, herpesvirus, AIV and *Brucella* spp, via polymerase chain reaction (PCR).

**Results:**

In northern Norway, cetacean morbillivirus, identified as the dolphin morbillivirus (DMV) strain, was detected in the blows of two asymptomatic groups of humpback whales, in the blow of one sperm whale in poor health and in the kidney of a stranded pilot whale. An alphaherpesvirus was detected in the blows of five humpback whale groups sampled in Norway, Iceland, and Cape Verde, while a gammaherpesvirus was detected in one humpback whale skin biopsy, sampled in Norway. No other samples tested positive to any of the pathogens, including AIV or *Brucella* spp.

**Conclusion:**

Our results demonstrate that minimally invasive sampling, particularly blow sampling, can be used for pathogen surveillance in free-ranging cetaceans. They also provide new insights into the circulation of cetacean morbillivirus and herpesviruses in cetaceans from the NE Atlantic. Continuous monitoring of pathogen exposure, alongside other stressors, will be crucial to assess the cumulative health implications for these cetaceans.

**Supplementary Information:**

The online version contains supplementary material available at 10.1186/s12917-025-05152-6.

## Background

Knowledge on pathogen prevalence in free-living large whales remains limited in the Northeast (NE) Atlantic, particularly in the Arctic and sub-Arctic regions [1, 2]. Historically, most data on pathogens results from sampling of dead stranded animals, but this approach is often limited by carcass decomposition levels, low detection and reporting rates, and lack of availability of experienced personnel [3–6]. Thus, monitoring free-swimming individuals through minimally invasive techniques offers a powerful complement to postmortem investigations [7–9].

Recently, exhaled breath or “blow” samples have been collected with poles or uncrewed aerial vehicles (UAVs, “drones”) and used to screen for pathogens such as cetacean morbillivirus [7, 10], avian influenza virus (AIV) and *Brucella* spp. [10]. These samples have also been used to describe the respiratory microbiome of multiple cetacean species [10–15]. On the other hand, biopsy sampling of skin and blubber is still the most routinely used technique to collect samples from free-swimming cetaceans [9]. However, while pathogens such as cetacean morbillivirus [16, 17], herpesvirus [18, 19] and poxvirus [20] have been detected in skin lesions from stranded individuals, the feasibility of using remotely collected biopsy samples for screening of these pathogens has not yet been thoroughly investigated.

Assessing and monitoring the full range of hosts and pathogens in a cetacean population is not feasible, especially when baseline data are scarce. Thus, a newly created pathogen surveillance program should first identify key pathogens and indicator species to be prioritized [21–25].

Cetacean morbillivirus, herpesvirus, *Brucella* spp., and AIV are amongst the most significant pathogens affecting cetaceans globally, and have been associated with severe disease and death, mostly through affecting the respiratory and neurological systems [1, 23, 26–28]. Cetacean morbillivirus, in particular, has caused several outbreaks of disease worldwide, and is often associated with profound immunosuppression [29]. As a result, individuals who survive the initial infection may still suffer from increased susceptibility to secondary pathogens [29, 30]. In this context, herpesviruses, which are often asymptomatic in healthy animals, can become opportunistic and cause mild to fatal disease in these immunocompromised hosts [26, 31–33]. Highly Pathogenic Avian Influenza Virus (HPAIV) is a zoonotic emerging pathogen of concern, responsible for recent outbreaks that have resulted in mass mortality of birds worldwide, but also affecting an increasing number of marine mammal species [34–40]. *Brucella* spp. is also considered zoonotic and, in addition to causing respiratory and neurological disease, can lead also to reproductive disorders in cetaceans, further impacting individual health and population dynamics [41].

The humpback whale (*Megaptera novaeangliae*) and sperm whale (*Physeter macrocephalus*) show an interesting potential as indicator species for pathogen surveillance in the NE Atlantic due to their migratory patterns, social structure and feeding aggregations, which may facilitate pathogen transmission [42–45]. NE Atlantic humpback whales follow predictable migratory routes between high latitude feeding grounds (Iceland, Greenland, Barents Sea) and tropical breeding areas (Cape Verde or Caribbean), with additional feeding stopovers in northern Norway (recently, off the town of Skjervøy) [42, 46]. Sperm whales present geographical sexual segregation, with females occurring year-round in maternal social units around low-latitude breeding grounds, and males migrating between these areas and high latitude feeding grounds (e.g. off Andenes, in northern Norway) [44, 47–50].

The high-density aggregations of cetaceans during feeding events may lead to interactions with other cetacean species, seabirds, and even humans, through whale-watching and snorkelling tourism [51]. Thus, these areas present a unique potential for the study of cetacean pathogen transmission dynamics, namely for the monitoring of pathogens that can be transmitted between cetaceans (e.g. morbillivirus, herpesvirus, *Brucella* spp.) [2, 31, 41], between cetaceans and birds (e.g. AIV) [34, 37], and between cetaceans and humans (e.g. *Brucella spp.*) [52]. Although no cases have been reported in the literature to date, AIV may also potentially be transmitted between cetaceans and humans due to its zoonotic risk [40].

The aim of this study was to survey four species of NE Atlantic cetaceans for selected pathogens of concern - cetacean morbillivirus, herpesvirus, *Brucella* spp., and AIV - using minimally invasive sampling of blow and skin. The study primarily targeted two migratory large whale species - the humpback whale and the sperm whale, but also reported findings from opportunistic samples collected from fin whales and a single stranded long-finned pilot whale.

## Materials and methods

### Blow and skin biopsy sampling

A consumer drone DJI Mavic 2 Pro, equipped with two foam floaters, low-noise propellers, and four sterilised Petri dishes, was used for the blow samples collection, according to the method described by Costa et al. (2023) [53]. The drone was flown between 0.5 and 3 m of altitude during sampling. To address the differences in the angles and shapes of the blow of the different species, the drone position was adapted according to the species being sampled. For humpback whales, the drone was flown above and behind the blowhole; for sperm whales it was flown above and to their left, as individuals of this species only possess a blowhole on the left side. After a successful sampling, the drone was returned to the vessel and the Petri dishes were dry-swabbed and placed in individual tubes with 1 ml of RNAlater (Thermo Fisher). The tubes were kept at ambient temperature during sampling (−5 °C to 6 °C), at 4 °C when back to land (for up to two weeks) and at −80 °C when back to the laboratory, until processing. When a group of animals was present (e.g. animals withing 5 m of each other), the sampling was recorded as group sampling, as cross-contamination of blows from multiple animals could not be avoided. Multiple sampling from the same individual or group was performed when there was an opportunity. Humpback and sperm whale flukes were photographed and used together with the drone footage to identify the different sampled individuals and to confirm there was no re-sampling [54]. Negative air controls were collected to account for environmental contamination, using the same methodology, but in the absence of any whales. Surface water samples (5 ml) were also collected in areas where whales were not present.

Biopsies were taken from the flank of individual animals, using an airgun (ARTS launching system, LKARTS-Norway) to deploy a floating arrow with a 4-cm long sterile stainless steel biopsy tip, cleaned between samplings (CetaDart) [43, 55]. Samples were stored at − 20 °C (before 2023) or −80 °C (from 2023 and forward), until analysis.

### Study areas, samples, and sampled individuals

**Fig. 1:**
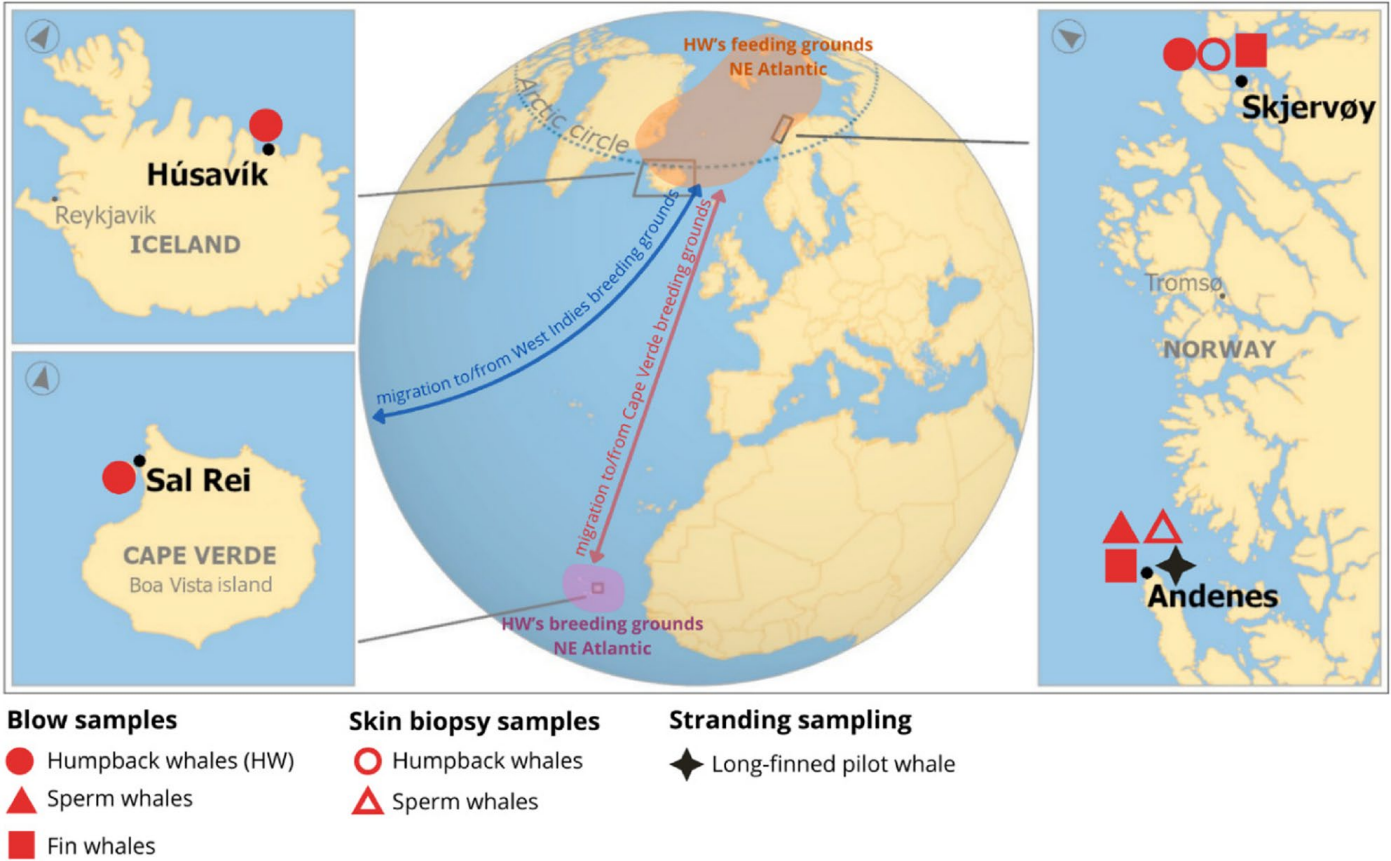
Overview of the study areas, types of samples collected and sampled species. Fieldwork locations are shown in bold. The humpback whale’s migration between the Northeast Atlantic feeding and breeding grounds is shown (arrows)

An overview of the study areas, sample types and sampled species is illustrated on Fig. 1 and an overview of collected samples is described in Table 1.

**Table 1:** Overview of collected samples

Species	Sample type	Date	Location	*n* whales sampled	*n* samples
*Humpback whale*	*Blow*	June 2022 December 2022 May 2023 December 2023 January 2025	Husavik, Iceland Skjervøy, Norway Sal Rei, Cape Verde Skjervøy, Norway Skjervøy, Norway	14 ind + 5 gr 15 gr 4 ind + 5 gr 2 ind + 4 gr 1 grp	19 17* 9 9* 1
	*Skin biopsy*	November 2016 November 2018 November 2020 November 2021 November 2023	Skjervøy, Norway Skjervøy, Norway Skjervøy, Norway Skjervøy, Norway Skjervøy, Norway	4 ind 3 ind 14 ind 4 ind 4 ind	4 3 14 4 4
*Sperm whale*	*Blow*	August 2023 March 2024	Andenes, Norway Andenes, Norway	8 ind 4 ind + 2 gr	13* 6
	*Skin biopsy*	2021 2022 2023 2024	Andenes, Norway Andenes, Norway Andenes, Norway Andenes, Norway	2 ind 5 ind 6 ind 3 ind	2 5 6 3
*Fin whale*	*Blow*	August 2023 January 2025	Andenes, Norway Skjervøy, Norway	1 ind 1 ind	1 1
*Pilot whale*	*Kidney* *Liver*	May 2024	Andenes, Norway	1 ind	1 1

ind= individuals; gr= groups (when more than one individual were present during sampling within 5 meters of each other)

*The same individual or group was sampled more than once. Additional information can be consulted in Supplementary Table 1

#### Blow samples

Between 2022 and 2025, a total of 55 blow samples were collected from 20 individuals and 30 groups of humpback whales along their North Atlantic migratory route. Sampling locations included the stopover feeding grounds in Skjervøy, northern Norway during winter; the breeding grounds in Sal Rei, Cape Verde, during spring; and the feeding grounds in Húsavík, Iceland, during summer (Fig. 1; Table 1). The groups of humpback whales ranged from two to 50 individuals (Supplementary Table 1).

Between 2023 and 2024, a total of 19 blow samples were collected from 12 individuals and 2 groups of sperm whales in Andenes, northern Norway, during summer and late winter (Fig. 1; Table 1). The groups of sperm whales had two animals (Supplementary Table 1).

Blow samples were opportunistically collected from two fin whales, one in Andenes, Norway, during summer and one in Skjervøy, Norway, during winter (Fig. 1; Table 1, Supplementary Table 1).

Due to the cross contamination of blows, the actual number of sampled individuals per group is unknown, and the number of positive animals per group was not possible to calculate. Thus, the prevalence of each pathogen was calculated based on sampled units, where both groups and individuals are considered as single sampling units.

#### Skin biopsies

Between 2016 and 2024, 29 skin biopsy samples were collected from humpback whales in Skjervøy, Norway. Between 2021 and 2024 16 skin biopsies were collected from sperm whales in Andenes, Norway (Fig. 1; Table 1; Supplementary Table 1).

#### Cases of interest

All individuals were travelling or feeding during sampling and did not display any uncommon behaviours or clinical signs, with the exception of a sperm whale and a long-finned pilot whale, both sampled in spring 2024, in Andenes (Fig. 1).

**Fig. 2:**
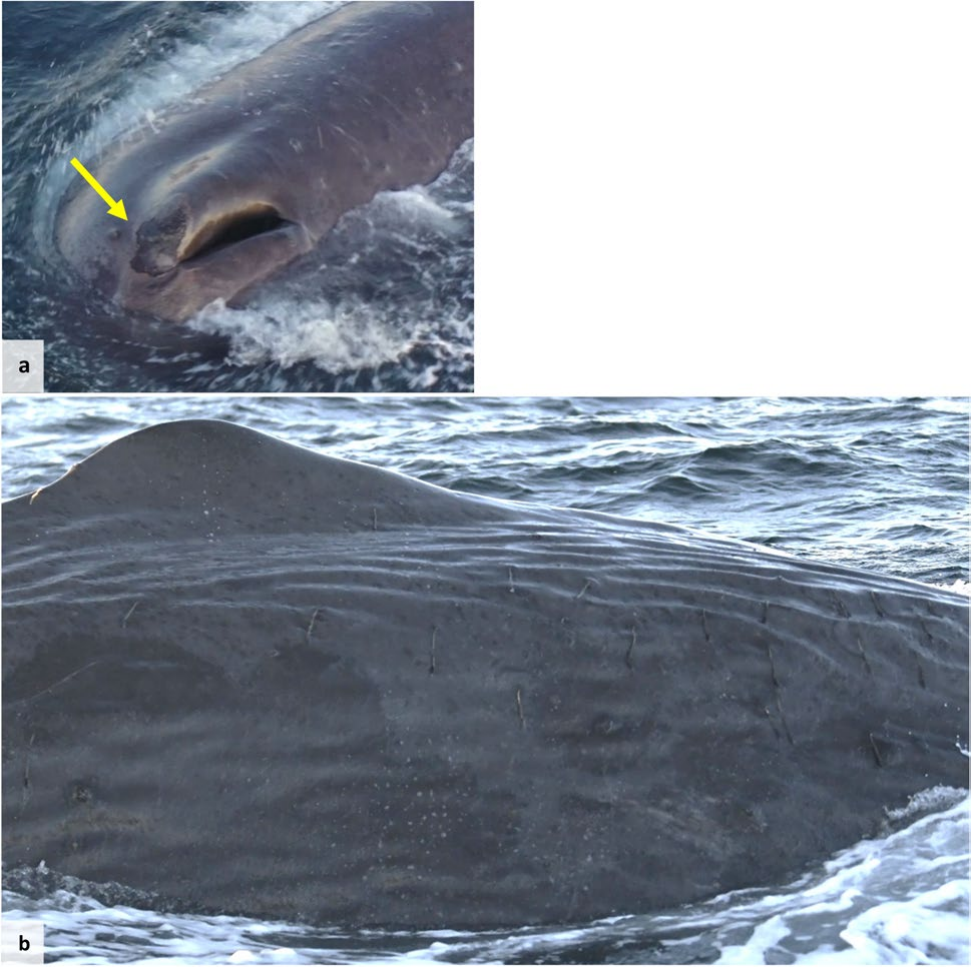
Sperm whale presenting a skin lesion cranially to the blowhole (**a**), indicated by a yellow arrow, and a high burden of skin parasites (**b**) (photograph credits (**a**) Helena Costa and (**b**) Zoë Morange)

In March 2024, a male sperm whale presented skin lesions (Fig. 2A) and a high burden of skin parasites, morphologically compatible with *Pennella* sp. [56] (Fig. 2B). Through the drone footage, the whale was estimated to measure 14.11 m long using MorphoMetriX software and the boat as reference [57]. While his body condition score was not calculated, the animal did not appear visually underweight. Dives were noted to be short and shallow for the species. The fluke was matched to the local sperm whale photo-identification catalogue and identified as a male previously sighted in the area in 2021 (Morange, personal observation). Despite the presence of gatherings of sperm whales at the time, this whale was sighted alone. Both a blow sample and a biopsy sample were collected from this individual (Supplementary Table 1).

**Fig. 3:**
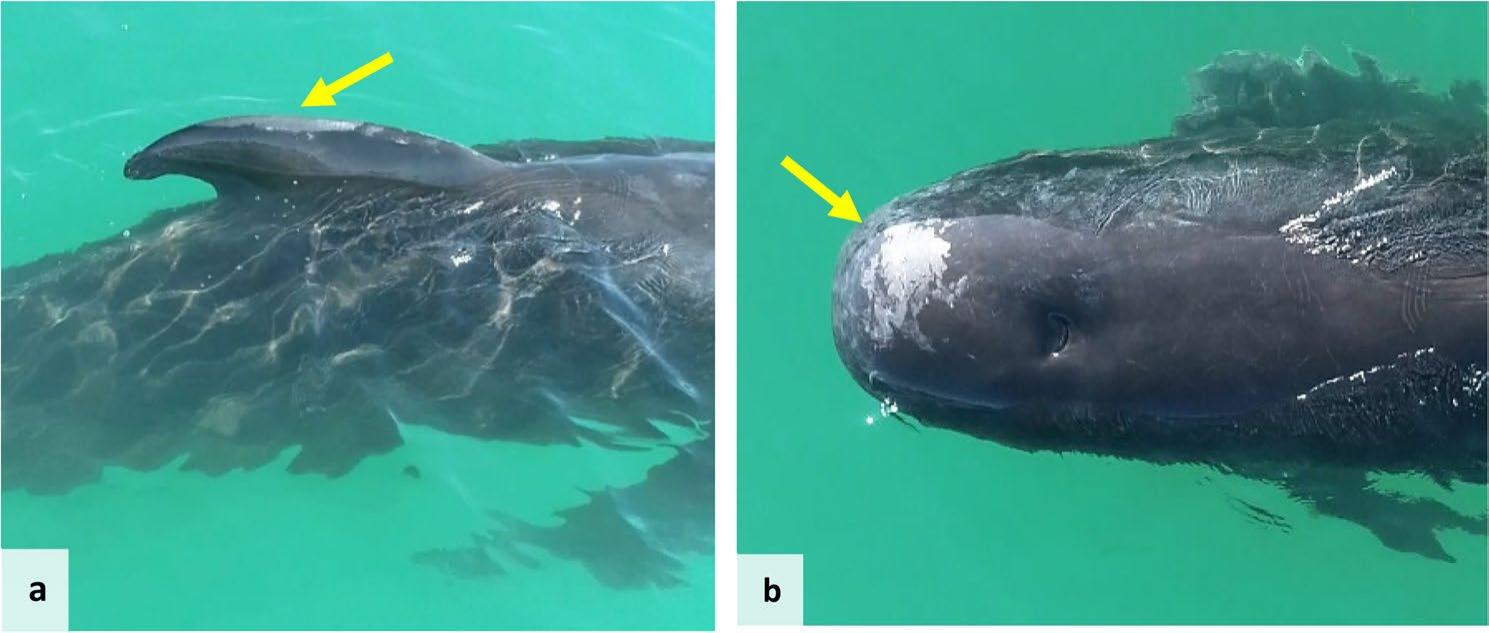
Long-finned pilot whale drone photographed while alive, and previously to stranding, presenting skin lesions on dorsal fin (**a**) and cranially to blowhole (**b**), indicated by yellow arrows (photographs credit Ricard Karoliussen)

In May 2024, an adult male long-finned pilot whale was sighted alone, in close proximity to the coast of Andenes, struggling with buoyancy, swimming sideways and in circles. Further, it also presented skin lesions on the dorsal fin and cranially to the blowhole (Fig. 3). A blow sample was collected, but did not pass the reference gene control (described below) and was not used for further analysis. In the next five hours, the whale stranded alive. Efforts were made to refloat the animal, but the operation was unsuccessful. The animal died six hours later, and a field necropsy was performed the next day (10 hours after death). Samples of liver and kidney were collected and kept in RNAlater at room temperature until analysis.

### Nucleic acids extraction

Nucleic acids were extracted from all blow samples and control samples using the HighPrep Viral/Pathogen DNA/RNA Kit (Magbio). DNA extraction from the skin samples was performed using the Dneasy Blood & Tissue Kit (QIAGEN). DNA concentration of all samples was measured on NanoDrop One and ran on an agarose gel for quality check. RNA extractions from the biopsies were performed using the PureLink RNA Mini Kit (Thermofisher). cDNA synthesis was performed using 6 µL of extracted sample and the Quantitect Reverse Transcriptase kit (Qiagen). To verify the presence of amplifiable RNA in the samples, a SYBR-green RT-qPCR targeting the reference gene YWHAZ was performed, as previously described for cetacean blow samples [7]. Each reaction was performed with the PowerTrack SYBR Green Master Mix kit (Applied Biosystems), with 250 nM of each primer (Supplementary Table 2), and 2 µL of cDNA. A positive, a non-RT control, and a non-template control (NTC) were included. Only samples with an amplifiable reference gene were used for further analysis. All methods were performed according to the manufacturer’s protocols.

### Cetacean morbillivirus, herpesvirus, Brucella spp. and avian influenza virus PCRs

A SYBR-green RT-qPCR targeting the CeMV phosphoprotein (P) gene was carried out to analyse the presence of morbillivirus RNA in all blow samples (*n* = 76), in the skin samples stored at −80 °C (*n* = 12), and in the pilot whale kidney and liver samples, using primers described previously (Supplementary Table 2) [58]. Each reaction was performed with the PowerTrack SYBR Green Master Mix kit (Applied Biosystems), with 300 nM of each primer (Supplementary Table 2), and 2 µL of cDNA. Amplification was carried out using Roche Diagnostics, LightCycler^®^ 96 System, as following: 95 °C for 2 min, 40 amplification cycles of 95 °C for 5 s and 59 °C for 1 min, followed by a melt curve analysis.

A nested pan-herpesvirus conventional PCR was carried out to analyse the presence of herpesvirus DNA in blow (*n* = 74) and all skin samples (*n* = 45), using degenerate primers targeting three highly conserved motifs of the viral DNA polymerase (DPOL) gene, as described previously (Supplementary Table 2) [59]. The PCR reaction was performed using AmpliTaq Gold 360 Master Mix (Applied Biosystems), with primers in a concentration of 400nM (Supplementary Table 2), and 50 ng of nucleic acid sample. Amplification was carried out using BIO-RAD C1000 Touch Thermo Cycler, as following: 94 °C for 30 s, 46 °C for 1 min, 72 °C for 1 min, followed by a final step of 72 °C for 7 min. The second reaction was performed under the same conditions, using 2.5 µL PCR product from the first reaction. Secondary PCR products were analysed on a 1.2% agarose gel and visualized using BIORAD ChemiDoc MP imaging system.

A one-step RT-qPCR was carried out to analyse the presence of AIV RNA in blow samples (*n* = 76) using primers targeting the matrix (M) gene, as described previously [60]. The PCR reaction was performed using qScript XLT 1-Step RTqPCR ToughMix (Qiagen), primers and probe in a concentration of 400 nM and 200nM, respectively (Supplementary Table 2), and 3 µL of nucleic acid sample. Amplification was carried out using Roche Diagnostics, LightCycler^®^ 96 System, as following: 50 °C for 10 min, 95 °C for 1 min and 45 amplification cycles of 95 °C for 10 s and 52 °C for 30 s.

Due to limited amount of material, only a portion of the blow samples (*n* = 18) were run for *Brucella* spp. A SYBR-green real-time PCR was carried out to analyse the presence of Brucella DNA using primers targeting the IS711 gene as previously described [61]. Each reaction was performed with the PowerTrack SYBR Green Master Mix kit (Applied Biosystems), with 300 nM of each primer (Supplementary Table 2) and 2 µL of DNA. Amplification was carried out using Roche Diagnostics, LightCycler^®^ 96 System, as following: 95 °C for 2 min, 40 amplification cycles of 95 °C for 5 s and 60 °C for 1 min, followed by a melt curve analysis.

All air and seawater control samples were processed in the same manner and confirmed negative for all pathogens. Positive (synthetic oligonucleotides, gBlock [62]) and non-template controls (RNAse-free water) were included in each reaction. All methods were performed according to the manufacturer’s protocols.

### Sequencing of positive samples and phylogenetic analysis

The PCR products of the positive samples were cleaned using ExoSAP-IT PCR Product Cleanup Reagent (Applied Biosystems) and both strands sequenced by the Sanger method. Consensus sequences were edited using MEGA11 software [63] and compared with those available on GenBank.

The cetacean morbillivirus sequences obtained in this study were aligned with other available sequences of the morbillivirus P gene retrieved from GenBank, using ClustalW [64] algorithm through the software MEGA 11 [63]. The herpesvirus sequences obtained in this study were translated to the deduced amino acid sequences and aligned to other available sequences of the herpesvirus DPOL gene obtained from cetacean hosts, retrieved from GenBank, using ClustalW [65] algorithm through the software MEGA 11 [63].

These alignments were used for the construction of two Maximum Likelihood phylogenetic trees, carried out through the IQ-TREE web platform (http://iqtree.cibiv.univie.ac.at) [65, 66]. The substitution models were determined based on the ModelFinder, which automatically uses Bayesian Information Criterion to select the best-fit model for each analysis [67]. Node support statistics were assessed using ultrafast bootstrapping with 1000 replicates [68]. A sequence of a Peste des petits ruminants virus (PPRV) from a sheep (*Ovis aries*) was used as an outgroup to root the cetacean morbillivirus phylogram and a sequence of a phocine herpesvirus from a harbor seal (*Phoca vitulina*) was used as an outgroup to root the herpesvirus phylogram. The resulting phylogenetic trees were visualized and refined using R software.

## Results

### Cetacean morbillivirus

**Fig. 4:**
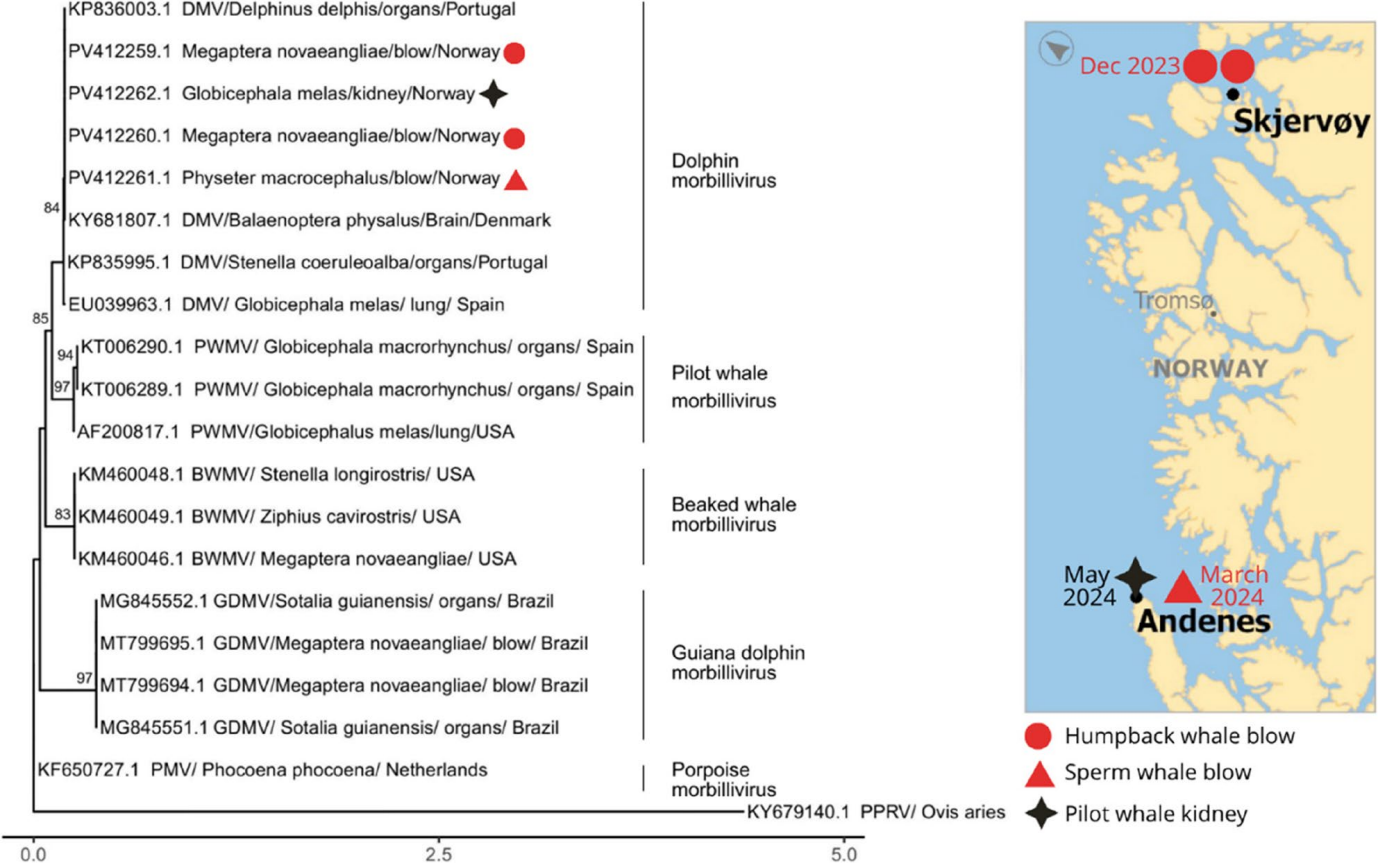
Cetacean morbillivirus positive samples (red circles, red triangle and black cross). A maximum-likelihood phylogenetic tree was inferred using nucleotide sequences of the morbillivirus phosphoprotein gene, representing relationships among the morbillivirus sequences obtained from this study and sequences obtained from other cetacean hosts retrieved from GenBank (left). The reliability of the tree topology was tested by bootstrapping 1000 replicates, and results are indicated at the tree nodes. Sequences are labelled as “*GenBank accession number – host* – *tissue where the sequence was obtained from* – *country*”. The scale bar represents genetic distance, measured as the number of nucleotide substitutions per site. Map illustrating general location (off Skjervøy or Andenes), dates, species and sample type of morbillivirus positive cases from this study (right)

Cetacean morbillivirus was detected in the blow samples from two groups of humpback whales in the winter of 2023, in Skjervøy; in the blow from one sperm whale in the spring of 2024, in Andenes; and in the kidney sample from a stranded pilot whale in the spring of 2024, in Andenes (Fig. 4; Supplementary Table 3). All samples collected prior to the winter 2023 tested negative for cetacean morbillivirus. All skin biopsy samples tested negative for cetacean morbillivirus, including the skin biopsy from the sperm whale with a positive blow (Supplementary Table 1, Supplementary Table 3).

Sequencing showed that the obtained sequences of the morbillivirus P gene were identical between each other and the BLAST analysis indicated that they were most closely related to multiple GenBank sequences of the dolphin morbillivirus (DMV) strain, obtained from NE Atlantic cetaceans, namely a stranded fin whale in 2016 in Denmark (KY681807.1). Accordingly, the phylogenetic analysis showed that the sequences obtained in this study clustered with sequences from the DMV strain obtained from other Atlantic cetaceans, both baleen and toothed whales. Further, the tree presented different clusters differentiating between the recognized strains of cetacean morbillivirus: dolphin morbillivirus (DMV), pilot whale morbillivirus (PWMV), beaked whale morbillivirus (BWMV), porpoise morbillivirus (PMV) and Guiana dolphin morbillivirus (GDMV) (Fig. 4).

### Herpesviruses

Herpesviruses were detected only in humpback whales, including in the blow samples from two groups collected in Skjervøy in 2023; two groups of whales collected in Cape Verde in 2023; one group of whales collected in Iceland in 2022; and in one skin sample collected in Skjervøy in 2020 (Supplementary Table 1; Supplementary Table 3; Supplementary Fig. 1). The two groups sampled in Skjervøy in 2023 that tested positive for herpesvirus were also the two groups that tested positive for cetacean morbillivirus (Supplementary Table 1).

**Fig. 5:**
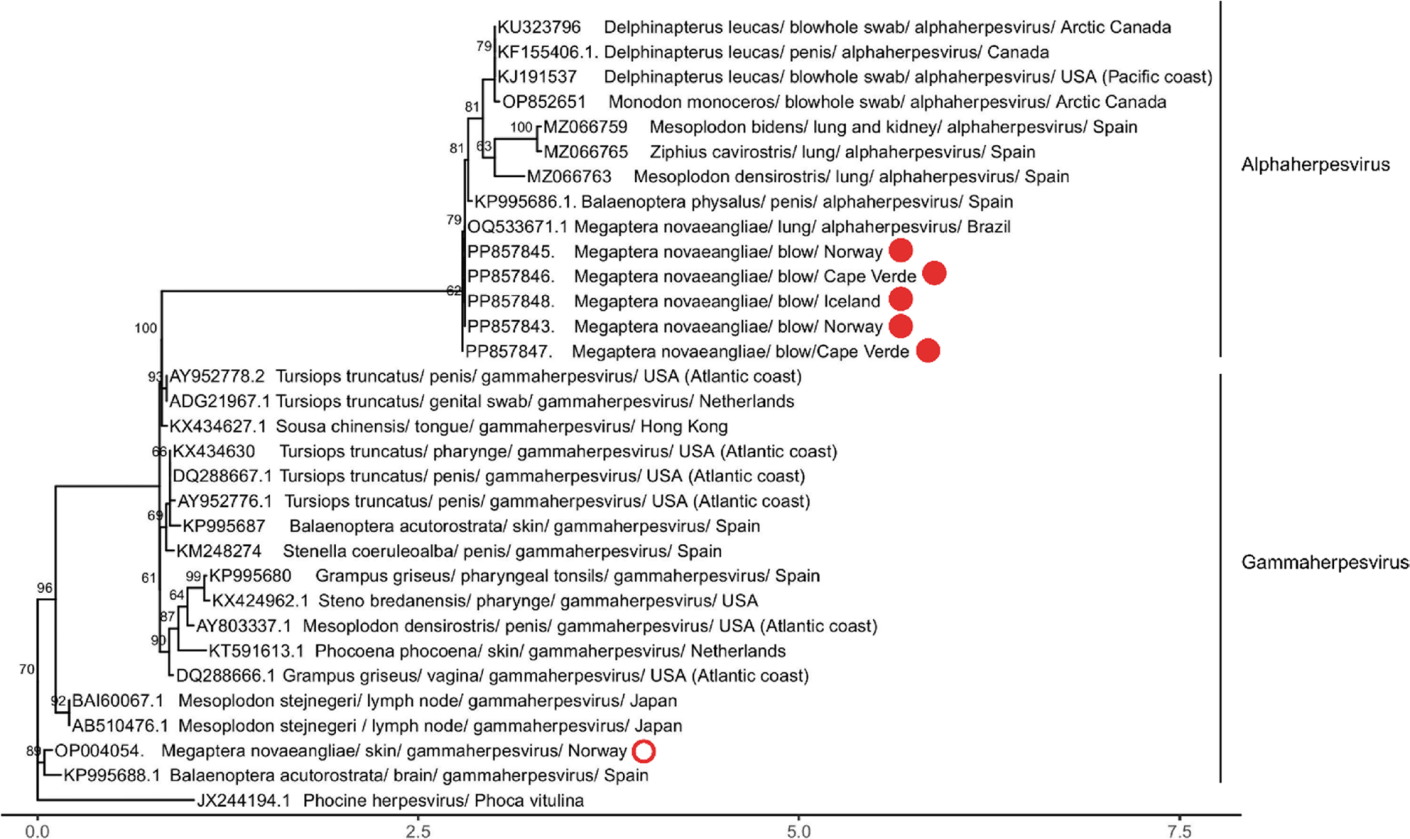
Herpesvirus positive samples (red circles). A maximum-likelihood phylogenetic tree was inferred using the amino acid sequences encoded by the herpesvirus DNA polymerase gene, representing relationships among the herpesvirus sequences obtained from the humpback whales’ blows (full red circle) and skin (red circle line) in this study and sequences obtained from other cetacean hosts retrieved from GenBank. The reliability of the tree topology was tested by bootstrapping 1000 replicates, and results are indicated at the tree nodes. Sequences are labelled as “*GenBank accession number – host* – *tissue where the sequence was obtained from* – *country*”. The scale represents genetic distance

The BLAST analysis showed that the amplified herpesvirus DPOL gene sequence obtained from the humpback whale skin was most similar to the GenBank sequences KP995688.1, a gammaherpesvirus obtained from the brain of a minke whale (*Balaenoptera acutorostrata)* stranded in the Mediterranean in 2014, with 80.84% identity. The herpesvirus DPOL gene sequences obtained from the blows of the humpback whales were identical between each other and the BLAST analysis showed that they were most similar to the GenBank sequence OQ533671.1, an alphaherpesvirus obtained from the lung of a humpback whale in Brazil with a 99.46%. Accordingly, the phylogenetic analysis showed that the sequence obtained from the skin clustered with sequences from the G*ammaherpesvirinae* subfamily and that the sequences obtained from the blows clustered with other sequences from the *Alphaherpesvirinae* subfamily. Further, while the tree presented a general clustering dividing strains obtained from baleen whales and among strains obtained from toothed whales, there were exceptions (Fig. 5).

### Avian influenza virus and *Brucella* spp.

All samples tested negative for AIV and *Brucella* spp. (Supplementary Table 1, Supplementary Table 3).

## Discussion

In this study, we have detected cetacean morbillivirus in three different cetacean species in Norway, and herpesviruses in humpback whales in Iceland, Cape Verde and Norway. No AIV or *Brucella* spp. were detected in any of the sampled species or locations.

### Cetacean morbillivirus

In the period of six months, between 2023 and 2024, cetacean morbillivirus of the strain DMV was detected in northern Norway in the blow of two asymptomatic groups of humpback whales, and of a sperm whale, as well as in the kidney of a stranded long-finned pilot whale.

Despite being reported globally, cetacean morbillivirus had never been reported in areas above the Arctic Circle. No protective antibodies were detected in white whales (*Delphinapterus leucas*) [69, 70] or narwhals (*Monodon monoceros*) [70] sampled in Arctic areas of Norway and Canada. Regardless, cetacean morbillivirus has been widely reported in the North Atlantic, including migratory species that occur seasonally in Arctic feeding grounds, such as fin whales [71, 72]. Thus, the lack of reported cases in the Arctic may be more reflective of historical gaps in surveillance, rather than representative of a true absence of the virus in the region.

Studies of morbillivirus in the NE Atlantic cetaceans report overall prevalences of 5.7% [73], while in the Mediterranean, prevalences have been reported up 31.9% [74]. However, these studies were based on molecular screening of multiple tissues from dead individuals, which limits direct comparisons with the results obtained here. For more comparable data, the virus was detected in blow samples from 4.17% of southern Atlantic humpback whale groups [7]. Accordingly, our results align closely with those findings, as we observed a 4% prevalence in the blow samples collected from humpback whales. However, considering our positive groups had three to 15 individuals, the exact number of infected individuals in each group could not be confirmed, making it possible that the real viral prevalence was higher.

The two positive humpback whale groups in our study were traveling or feeding, and no abnormal behavior or clinical signs were noted. While the impact of this morbillivirus infection on the health of these whales remains uncertain, previous research on stranded humpback whales has shown that this virus has the potential to be highly pathogenic to the species, especially among younger or more vulnerable individuals [75, 76].

Conversely, the cetacean morbillivirus-positive sperm and pilot whales in our study showed signs of poor health, including high burden of skin parasites morphologically compatible with *Pennella* sp., cutaneous lesions, and abnormal diving behaviours. While *Pennella* sp. infestations are typically mild, heavy ones have been linked to poor health and to morbilliviral infections [57, 77]. Further, the abnormal buoyancy shown by both animals may be potentially related to respiratory disease caused by the virus [78, 79]. Although cetacean morbillivirus can cause cutaneous lesions [16], no skin biopsy sample tested positive to the virus. On the other hand, poxviruses could also have caused these lesions [20]. Thus, further research is needed to better understand the aetiology of these lesions, including screening for additional pathogens such as poxviruses. However, it’s important to note that the method of remote skin biopsying is not able to specifically target skin lesions in localized areas, i.e. around the blowhole. Thus, this technique might present limited diagnostic value for localized viral infections.

Overall, further studies are necessary to understand whether cetacean morbillivirus was the main cause of disease, a contributing factor, or if the whales were just carriers of the virus. Therefore, we recommend a continued monitoring for this virus in live and stranded cetaceans in northern Norway, to further investigate prevalence, effects, co-infections and host-susceptibilities.

### Herpesviruses

An alphaherpesvirus was detected in blow samples from five humpback whale groups, throughout different years and locations, while a gammaherpesvirus was detected in the skin sample of a humpback whale from northern Norway. A high prevalence of herpesviruses has been detected by PCR in stranded odontocetes in the Mediterranean Sea and NE Atlantic [31, 80, 81], which are often considered not clinically significant on their own. While no studies have reported herpesviruses prevalence in blow or skin biopsy samples, the prevalence of this virus in the respiratory system and integument of odontocetes stranded in the Mediterranean was 6.78% and 14.40%, respectively [31]. In our study, we detected the virus in the blow of five out of 49 groups of humpback whales sampled, with a prevalence of 10.20%; and in the skin biopsy of one out of 29 groups of humpback whales sampled, with a prevalence of 3.45%. The difference in prevalences between studies might be related to the different species sampled; geographically related; or related to the different methodologies used for sampling.

The two humpback whale groups that tested positive for cetacean morbillivirus in northern Norway also tested positive for herpesvirus. Co-infections of cetacean morbillivirus with herpesvirus have been reported multiple times in multiple cetacean species [31, 32, 80, 82]. This can be explained by the fact that animals that survive the acute stage of morbillivirus infection are more susceptible to opportunistic infections due to immunosuppression caused by the virus [29]. However, since the sampled groups had between 3 and 15 animals, we cannot confirm with certainty if the detected viruses had originated in the same individuals.

### Avian influenza virus

No samples in this study tested positive for AIV. Case reports of AIV in cetaceans are still few and include a bottlenose dolphin (*Tursiops truncatus*) in Florida in 2022 [37] and a porpoise (*Phocoena phocoena*) in Sweden 2022 [34]. In the summer of 2023, HPAIV (H5N5) was detected in one dead walrus (*Odobenus rosmarus rosmarus*) in Svalbard [39]. In 2025, the virus (H5N7) was detected in dead birds in the fjord off Skjervøy, where the whale winter-feeding aggregations take place [83]. Considering this is a zoonotic virus, tourists that snorkel and dive in the area might be in high risk of getting infected. Thus, considering the current significant threat that the virus is causing to wild birds and other mammals, and the close proximity between cetaceans, birds and humans during feeding aggregations, a continued surveillance for this pathogen in the area is warranted.

### *Brucella* spp.

No *Brucella* spp. was detected in any samples in this study. Antibodies anti-*Brucella* spp. and *Brucella* DNA have been detected in cetaceans sampled in the North Atlantic [70, 84–86]. Overall, while *Brucella* spp. seems to be widespread in the region, the relatively low antibody prevalence detected in baleen whales [84] can explain the lack of detection of active infection in the 17 samples we screened in this study, mostly collected from humpback whales. However, because *Brucella* spp. is considered zoonotic and can cause reduced reproductive success in its hosts, among other detrimental health effects [41], continued surveillance for this pathogen also remains crucial.

## Conclusions

To our knowledge, this study includes: (1) the first molecular report of cetacean morbillivirus in Norway and above the Arctic Circle, (2) the first report of blow sampling for pathogen surveillance in sperm and fin whales, and (3) the first study exploring the use of remotely collected biopsy samples for viral screening. With our findings, we demonstrate the suitability of using drone blow sampling as a non-invasive tool for pathogen surveillance in free-ranging whales and provide new insights into the circulation of cetacean morbillivirus and herpesviruses in cetaceans from the NE Atlantic. Overall, continuous monitoring of pathogen exposure, alongside other stressors, will be essential for understanding disease dynamics, assessing possible health implications, and helping to guide future conservation strategies for these and other cetaceans occurring in the North Atlantic.

## Supplementary Information

Supplementary Material 1.

## Data Availability

All data generated or analysed during this study are included in this published article and its supplementary information files.
