# Supplementary material for: Deep breath out: molecular survey of selected pathogens in blow and skin biopsies from North Atlantic cetaceans
Source: BMC Vet Res. 2025 Dec 18;21:709. doi: 10.1186/s12917-025-05152-6 (PMC12713300; doi:10.1186/s12917-025-05152-6)
Supplement: Supplementary file 1 — Supplementary Material 1. [file 12917_2025_5152_MOESM1_ESM.docx]

**Supporting information for**

**Deep breath out: molecular survey of selected pathogens in blow and skin biopsies from North Atlantic cetaceans**

Helena Costa^1,*^, Per Ramstedt^1,2^, Myrthe Bergsma^1^, Eve Jourdain^3^, Zoë Morange^2^, Pierre Blévin^4^, Charla J. Basran^5^, Marianne Rasmussen^5^, Terence P. Dawson^6^, Harriet Y. Dawson^7^, Seán A. O’Callaghan^8^, Prabhugouda Siriyappagouder^1^, Jorge M. O. Fernandes^1,9^, Audun H. Rikardsen^2,10^, Courtney A. Waugh^1^

^1^ Faculty of Biosciences and Aquaculture, Nord University, Universitetsalléen 11, 8026 Bodø, Norway

^2^ Department of Arctic and Marine Biology, UiT The Arctic University of Norway, Hansine Hansens veg 18, 9019 Tromsø

^3^ Norwegian Orca Survey, Breivikveien 10, Andenes NO-8480, Norway

^4^ Akvaplan-niva AS, Fram Centre, Hjalmar Johansens gate 14, 9007 Tromsø, Norway

^5^ University of Iceland, Húsavík Research Centre, Hafnarstétt 3, 640 Húsavík, Iceland

^6^ Department of Geography, King’s College London, Strand, London WC2R 2LS, UK

^7^ The Royal (Dick) School of Veterinary Studies, The University of Edinburgh, Easter Bush Campus, Midlothian EH25 9RG, UK

^8^ Marine and Freshwater Research Centre, Atlantic Technological University Galway City, Old Dublin Road, Galway, H91 T8NW, Ireland

^9^ Department of Renewable Marine Resources, Institute of Marine Sciences (ICM-CSIC), 08003 Barcelona, Spain

^10^ Norwegian Institute of Nature Research, Fram Centre, Hjalmar Johansens gate 14, 9007 Tromsø, Norway

* corresponding author: helena.g.costa@hotmail.com

**Supplementary Table 1.** Species, sample type, sample ID, date, location, and PCR results. Neg.= negative; Pos.= positive.

| Species | Sample type | Group/  individual ID | Sample ID | Nr of animals | Date | Location | CeMV | HV | *Brucella* spp. | AIV | Observations |
| --- | --- | --- | --- | --- | --- | --- | --- | --- | --- | --- | --- |
| Humpback whale | *Blow* | HW1  HW2  HW3  HW4  HW5  HW6  HW7  HW8  HW9  HW10  HW11  HW12  HW13  HW14  HW15  HW16  HW17  HW18  HW 19  HW20  HW21  HW22  HW23  HW24  HW25  HW26  HW27  HW28  HW29  HW30  HW31  HW32  HW33  HW34  HW35  HW36  HW37  HW38  HW39  HW40  HW41  HW42  HW43  HW44  HW45  HW46  HW47  HW48  HW49  HW50 | HW-1-ic  HW-2-ic  HW-3-ic  HW-4-ic  HW-5-ic  HW-6-ic  HW-7-ic  HW-8-ic  HW-9-ic  HW-10-ic  HW-11-ic  HW-12-ic  HW-13-ic  HW-14-ic  HW-15-ic  HW-16-ic  HW-17-ic  HW-18-ic  HW-19-ic  HW-20-no  HW-21-no  HW-22-no  HW-23-no  HW-24-no  HW-25-no A  HW-25-no B  HW-26-no  HW-27-no  HW-28-no  HW-29-no A  HW-29-no B  HW-30-no  HW-31-no  HW-32-no  HW-33-no  HW-34-no  HW-35-cave  HW-36-cave  HW-37-cave  HW-38-cave  HW-39-cave  HW-40-cave  HW-41-cave  HW-42-cave  HW-43-cave  HW-44-no  HW-45-no A  HW-45-no B  HW-46-no  HW-47-no  HW-48-no A  HW-48-no B  HW-49-no A  HW-49-no B  HW-50-no | 6  1  1  2  1  1  1  1  1  3  1  1  1  4  1  1  2  1  1  10-15  20-25  10  50  2  4-5  20  50  5  30  25  15  8  4  4  2  1  2  1  1  2  1  3  2  1  2  3  1  6  12-15  4 | Jun. 2022  Jun. 2022  Jun. 2022  Jun. 2022  Jun. 2022  Jun. 2022  Jun. 2022  Jun. 2022  Jun. 2022  Jun. 2022  Jun. 2022  Jun. 2022  Jun. 2022  Jun. 2022  Jun. 2022  Jun. 2022  Jun. 2022  Jun. 2022  Jun. 2022  Dec. 2022  Dec. 2022  Dec. 2022  Dec. 2022  Dec. 2022  Dec. 2022  Dec. 2022  Dec. 2022  Dec. 2022  Dec. 2022  Dec. 2022  Dec. 2022  Dec. 2022  Dec. 2022  Dec. 2022  Apr. 2023  Apr. 2023  Apr. 2023  Apr. 2023  Apr. 2023  Apr. 2023  Apr. 2023  Apr. 2023  Apr. 2023  Dec. 2023  Dec. 2023  Dec. 2023  Dec. 2023  Dec. 2023  Dec. 2023  Dec. 2023  Dec. 2023  Dec. 2023  Jan. 2025 | Húsavík  Húsavík  Húsavík  Húsavík  Húsavík  Húsavík  Húsavík  Húsavík  Húsavík  Húsavík  Húsavík  Húsavík  Húsavík  Húsavík  Húsavík  Húsavík  Húsavík  Húsavík  Húsavík  Skjervøy  Skjervøy  Skjervøy  Skjervøy  Skjervøy  Skjervøy  Skjervøy  Skjervøy  Skjervøy  Skjervøy  Skjervøy  Skjervøy  Skjervøy  Skjervøy  Skjervøy  Sal Rei  Sal Rei  Sal Rei  Sal Rei  Sal Rei  Sal Rei  Sal Rei  Sal Rei  Sal Rei  Skjervøy  Skjervøy  Skjervøy  Skjervøy  Skjervøy  Skjervøy  Skjervøy  Skjervøy  Skjervøy  Skjervøy | Neg.  Neg.  Neg.  Neg.  Neg.  Neg.  Neg.  Neg.  Neg.  Neg.  Neg.  Neg.  Neg.  Neg.  Neg.  Neg.  Neg.  Neg.  Neg.  Neg.  Neg.  Neg.  Neg.  Neg.  Neg.  Neg.  Neg.  Neg.  Neg.  Neg.  Neg.  Neg.  Neg.  Neg.  Neg.  Neg.  Neg.  Neg.  Neg.  Neg.  Neg.  Neg.  Neg.  Neg.  Neg.  Neg.  Neg  Neg.  **Pos.**  Neg.  Neg.  Neg.  **Pos.**  **Pos.**  Neg. | Neg.  Neg.  Neg.  Neg.  Neg.  Neg.  Neg.  Neg.  **Pos.**  Neg.  Neg.  Neg.  Neg.  Neg.  Neg.  Neg.  Neg.  Neg.  Neg.  Neg.  Neg.  Neg.  Neg.  Neg.  Neg.  Neg.  Neg.  Neg.  Neg.  Neg.  Neg.  Neg.  Neg.  Neg.  Neg.  Neg.  Neg.  Neg.  Neg.  **Pos.**  **Pos.**  Neg.  Neg.  Neg.  Neg.  Neg.  **Pos.**  Neg.  **Pos.**  Neg.  Neg.  Neg.  **Pos.**  **Pos.**  **-** | -  -  -  -  -  -  -  -  -  -  -  -  -  Neg.  -  -  Neg.  -  Neg.  Neg.  -  -  -  -  -  -  -  -  Neg.  -  -  -  Neg.  -  -  -  -  -  -  -  -  Neg.  -  Neg.  -  -  Neg.  Neg.  Neg.  Neg.  Neg.  -  Neg.  -  - | Neg.  Neg.  Neg.  Neg.  Neg.  Neg.  Neg.  Neg.  Neg.  Neg.  Neg.  Neg.  Neg.  Neg.  Neg.  Neg.  Neg.  Neg.  Neg.  Neg.  Neg.  Neg.  Neg.  Neg.  Neg.  Neg.  Neg.  Neg.  Neg.  Neg.  Neg.  Neg.  Neg.  Neg.  Neg.  Neg.  Neg.  Neg.  Neg.  Neg.  Neg.  Neg.  Neg.  Neg.  Neg.  Neg.  Neg.  Neg.  Neg.  Neg.  Neg.  Neg.  Neg.  Neg.  Neg. | -  -  -  -  -  -  -  -  -  -  -  -  -  -  -  -  -  -  -  -  -  -  -  Mother and juvenile  -  -  -  -  -  -  -  -  -  -  -  -  Mother and calf  Juvenile  Mother and calf  -  -  Mother and calf  -  Mother, calf, and escort  -  -  -  -  -  -  -  -  -  - |
|  | *Skin biopsy* |  | HW-1-S  HW-2-S  HW-3-S  HW-4-S  HW-5-S  HW-6-S  HW-7-S  HW-8-S  HW-9-S  HW-10-S  HW-11-S  HW-12-S  HW-13-S  HW-14-S  HW-15-S  HW-16-S  HW-17-S  HW-18-S  HW-19-S  HW-20-S  HW-21-S  HW-22-S  HW-23-S  HW-24-S  HW-25-S  HW-26-S  HW-27-S  HW-28-S  HW-29-S | 1  1  1  1  1  1  1  1  1  1  1  1  1  1  1  1  1  1  1  1  1  1  1  1  1  1  1  1  1 | 2016  2016  2016  2016  2018  2018  2018  2020  2020  2020  2020  2020  2020  2020  2020  2020  2020  2020  2020  2020  2020  2021  2021  2021  2021  2023  2023  2023  2023 | Skjervøy  Skjervøy  Skjervøy  Skjervøy  Skjervøy  Skjervøy  Skjervøy  Skjervøy  Skjervøy  Skjervøy  Skjervøy  Skjervøy  Skjervøy  Skjervøy  Skjervøy  Skjervøy  Skjervøy  Skjervøy  Skjervøy  Skjervøy  Skjervøy  Skjervøy  Skjervøy  Skjervøy  Skjervøy  Skjervøy  Skjervøy  Skjervøy  Skjervøy | -  -  -  -  -  -  -  -  -  -  -  -  -  -  -  -  -  -  -  -  -  -  -  -  -  Neg.  Neg.  Neg.  Neg. | Neg.  Neg.  Neg.  Neg.  Neg.  Neg.  Neg.  Neg.  Neg.  Neg.  Neg.  Neg.  Neg.  **Pos.**  Neg.  Neg.  Neg.  Neg.  Neg.  Neg.  Neg.  Neg.  Neg.  Neg.  Neg.  Neg.  Neg.  Neg.  Neg. | -  -  -  -  -  -  -  -  -  -  -  -  -  -  -  -  -  -  -  -  -  -  -  -  -  -  -  -  - | -  -  -  -  -  -  -  -  -  -  -  -  -  -  -  -  -  -  -  -  -  -  -  -  -  -  -  -  - | -  -  -  -  -  -  -  -  -  -  -  -  -  Seen with calf  -  -  -  -  -  -  -  -  -  -  -  -  -  -  - |
| Sperm whale | *Blow* | SW1  SW2  SW3  SW4  SW5  SW6  SW7  SW8  SW9  SW10  SW11  SW12  SW13  SW14 | SW-1-23 A  SW-1-23 B  SW-2-23 A  SW-2-23 B  SW-3-23 A  SW-3-23 B  SW-4-23  SW-5-23  SW-6-23 A  SW-6-23 B  SW-7-23 A  SW-7-23 B  SW-8-23  SW-9-23  SW-10-24  SW-11-24  SW-12-24  SW-13-24  SW-14-24 | 1  1  1  1  1  1  1  1  1  1  1  2  1  2 | Aug.2023  Aug.2023  Aug.2023  Aug.2023  Aug.2023  Aug.2023  Aug.2023  Aug.2023  Mar. 2024  Mar. 2024  Mar. 2024  Mar. 2024  Mar. 2024  Mar. 2024 | Andenes  Andenes  Andenes  Andenes  Andenes  Andenes  Andenes  Andenes  Andenes  Andenes  Andenes  Andenes  Andenes  Andenes  Andenes  Andenes  Andenes  Andenes  Andenes | Neg.  Neg.  Neg.  Neg.  Neg.  Neg.  Neg.  Neg.  Neg.  Neg.  Neg.  Neg.  Neg.  Neg.  **Pos.**  Neg.  Neg.  Neg.  Neg. | Neg.  Neg.  Neg.  Neg.  Neg.  Neg.  Neg.  Neg.  Neg.  Neg.  Neg.  Neg.  Neg.  Neg.  Neg.  Neg.  Neg.  Neg.  Neg. | -  -  -  -  -  -  -  -  -  -  -  -  -  -  Neg.  -  Neg.  Neg.  Neg. | Neg.  Neg.  Neg.  Neg.  Neg.  Neg.  Neg.  Neg.  Neg.  Neg.  Neg.  Neg.  Neg.  Neg.  Neg.  Neg.  Neg.  Neg.  Neg. | -  -  -  -  -  -  -  -  -  -  -  -  -  -  Individual with skin lesions and parasites.  -  -  -  - |
|  | *Skin*  *biopsy* |  | SW-1-S  SW-2-S  SW-3-S  SW-4-S  SW-5-S  SW-6-S  SW-7-S  SW-8-S  SW-9-S  SW-10-S  SW-11-S  SW-12-S  SW-13-S  SW-14-S  SW-15-S  SW-16-S | 1  1  1  1  1  1  1  1  1  1  1  1  1  1  1  1 | 2021  2021  2022  2022  2022  2022  2022  2023  2023  2023  2023  2023  2023  2024  2024  2024 | Andenes  Andenes  Andenes  Andenes  Andenes  Andenes  Andenes  Andenes  Andenes  Andenes  Andenes  Andenes  Andenes  Andenes  Andenes  Andenes | -  -  -  -  -  -  -  Neg.  Neg.  Neg.  Neg.  Neg.  Neg.  Neg.  Neg.  Neg. | Neg.  Neg.  Neg.  Neg.  Neg.  Neg.  Neg.  Neg.  Neg.  Neg.  Neg.  Neg.  Neg.  Neg.  Neg.  Neg. | -  -  -  -  -  -  -  -  -  -  -  -  -  -  -  - | -  -  -  -  -  -  -  -  -  -  -  -  -  -  -  - | -  -  -  -  -  -  -  -  -  -  -  -  -  -  Same as SW-10-24. Individual with skin lesions and parasites.  - |
| Fin whale | *Blow* | FW1  FW2 | FW-1-no  FW-2-no | 1  1 | Aug.2023  Jan. 2025 | Andenes  Skjervøy | Neg.  Neg. | Neg.  - | -  - | Neg  Neg | -  - |
| Pilot whale | *Liver*  *Kidney* | PW1 | PW-1-li  PW-1-kd | 1  1 |  | Andenes | Neg**.**  **Pos.** | Neg.  Neg. | -  - | Neg.  Neg. | -  - |

**Supplementary Table 2**. List of primers used. F= forward; R= reverse; P= probe.

| Gene | Primers | Annealing temp. | Reference |
| --- | --- | --- | --- |
| YWHAZ | F: 5'-GCAAAAGACGGAAGGTGCTG-3' |  | (Groch et al., 2021) |
|  | R: 5'-TGCTTGTGAAGCATTGGGGA-3' | 60° C |  |
| Morbillivirus phosphoprotein (P) | F: 5'-CCTCTAACAGGGGATCTRCTC-3' | 60° C | (Groch et al., 2020) |
|  | R: 5'-CCTGTGCCCTTTTTAATGGA-3' |  |  |
| Herpesvirus DNA polymerase (DPOL) | F: 5'-GAYTTYGCNAGYYTNTAYCC-3' (1^st^ reaction) | 46° C | (Vandevanter et al., 1996) |
|  | F: 5'-TCCTGGACAAGCAGCARNYSGCNMTNAA-3' (1^st^ reaction) |  |  |
|  | R: 5'-GTCTTGCTCACCAGNTCNACNCCYTT-3' (1^st^ reaction) |  |  |
|  | F: 5'-TGTAACTCGGTGTAYGGNTTYACNGGNGT-3' (2^nd^ reaction) |  |  |
|  | R: 5'-CACAGAGTCCGTRTCNCCRADAT-3' (2^nd^ reaction) |  |  |
| Brucella spp. IS711 | P: 5′-TACCGCTGCGAATAAAGCCAAC-3′ | 60° C | (Wu et al., 2014) |
|  | F: 5′-TGAGATTGCTGGCAATGAAGGC-3′ |  |  |
| AIV matrix (M) | F: 5'-AGATGAGTCTTCTAACCGAGGTCG-3’ | 52° C | (Spackman et al., 2003) |
|  | R: 5'-TGCAAAAACATCTTCAAGTCTCTG-3' |  |  |
|  | P: 5'- FAM-TCAGGCCCCCTCAAAGCCGA-TAMRA-3' |  |  |

**Supplementary Table 3.** Pathogen screening results by sample type, with the total number of positives sampled units in bold and the total number of screened sampled units in parentheses. Ic: Iceland; No: Norway; CaVe: Cape Verde.

| Species | Sample type | Date | Location | Cetacean morbillivirus | Herpesvirus | AIV | *Brucella* |
| --- | --- | --- | --- | --- | --- | --- | --- |
| *Humpback whale* | *Blow* | June 2022  December 2022  May 2023  December 2023  January 2025 | Husavik, Iceland  Skjervøy, Norway  Sal Rei, Cape Verde  Skjervøy, Norway  Skjervøy, Norway  ***Prevalence*** | 0 (19)  0 (15)  0 (9)  **2** (6)  0 (1)  ***2/50 = 4%*** | **1** (19)  0 (15)  **2** (9)  **2** (6)  -  ***5/49 = 10.2%*** | 0 (19)  0 (15)  0 (9)  0 (6)  0 (1)  - | 0 (3)  0 (3)  0 (2)  0 (5)  -  - |
|  | *Skin biopsy* | November 2016  November 2018  November 2020  November 2021  November 2023 | Skjervøy, Norway  Skjervøy, Norway  Skjervøy, Norway  Skjervøy, Norway  Skjervøy, Norway  ***Prevalence*** | 0 (4)  0 (3)  0 (14)  0 (4)  0 (4)  - | 0 (4)  0 (3)  **1** (14)  0 (4)  0 (4)  ***1/29 = 3.45%*** | -  -  -  -  -  - | -  -  -  -  -  - |
| *Sperm whale* | *Blow* | August 2023  March 2024 | Andenes, Norway  Andenes, Norway  ***Prevalence*** | 0 (8)  **1** (5)  ***1/15 = 7.69%*** | 0 (8)  0 (5)  - | 0 (8)  0 (5)  - | -  0 (4)  - |
|  | *Skin biopsy* | 2021  2022  2023  2024 | Andenes, Norway  Andenes, Norway  Andenes, Norway  Andenes, Norway | 0 (2)  0 (5)  0 (6)  0 (3) | 0 (2)  0 (5)  0 (6)  0 (3) | -  -  -  - | -  -  -  - |
| *Fin whale* | *Blow* | August 2023  January 2025 | Andenes, Norway  Skjervøy, Norway | 0 (1)  0 (1) | 0 (1)  - | 0 (1)  0 (1) | -  - |
| *Pilot whale* | *Blow*  *Kidney*  *Liver* | May 24 | Andenes, Norway | -  **1** (1)  0 (1) | -  0 (1)  0 (1) | -  0 (1)  0 (1) | -  -  - |

**
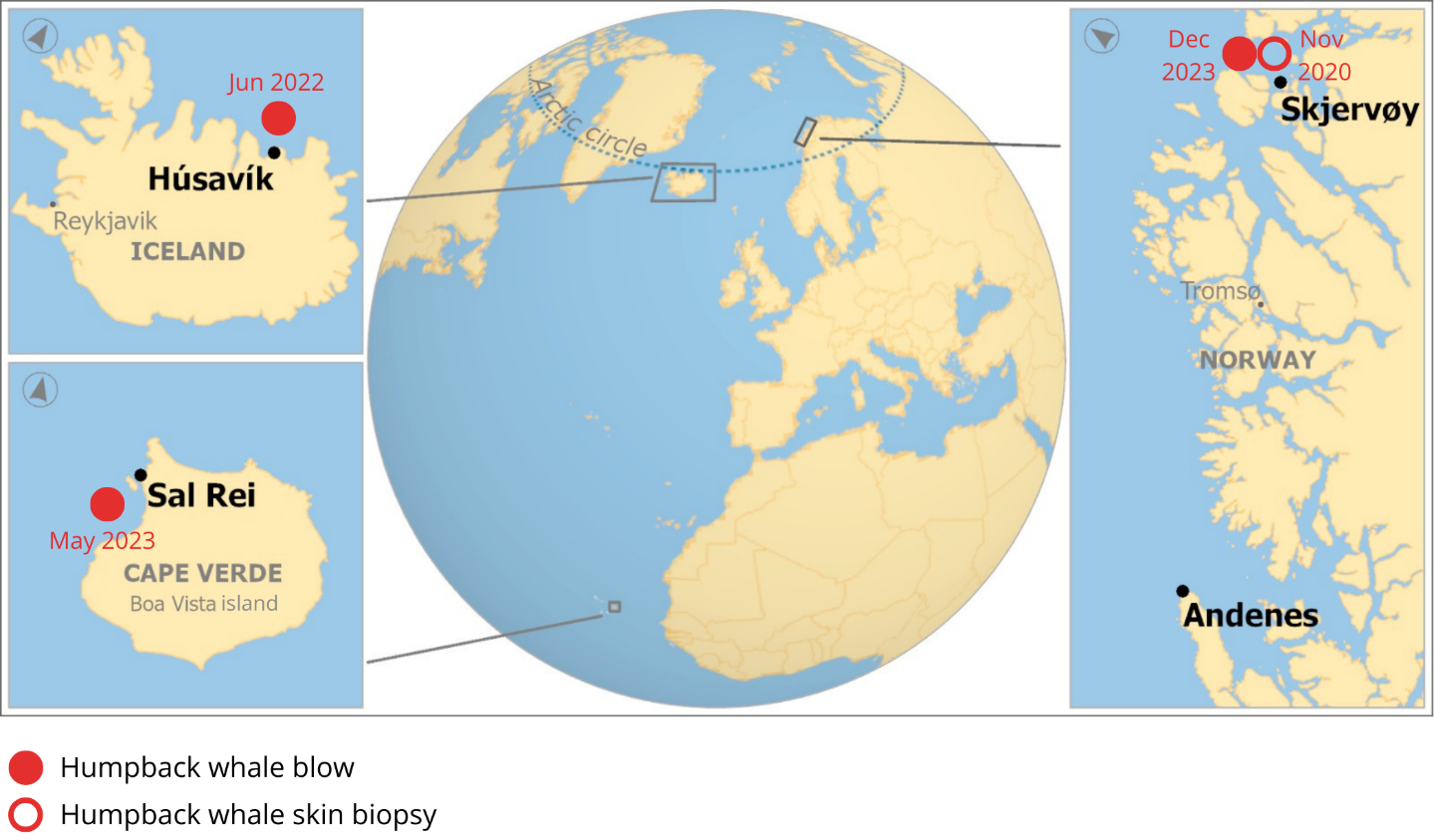
**

**Supplementary figure 1.** Map illustrating general locations (Iceland, Cape Verde or Norway/Skjervøy), dates, species and sample type of herpesvirus positive cases from this study.
